# Mapping the Theoretical Domain Framework to the Consolidated Framework for Implementation Research: do multiple frameworks add value?

**DOI:** 10.1186/s43058-023-00466-8

**Published:** 2023-08-24

**Authors:** B. O’Donovan, C. Kirke, M. Pate, S. McHugh, K. Bennett, C. Cahir

**Affiliations:** 1 Data Science Centre, School of Population Health, RCSI University of Medicine and Health Sciences, Dublin, Ireland; 2 Quality Improvement Division, Health Service Executive (HSE), Medication Safety, Dublin, Ireland; 3 School of Public Health, University College Cork, Cork, Ireland

**Keywords:** Consolidated framework for implementation research, Theoretical domains framework, Implementation frameworks, Medication safety

## Abstract

**Background:**

Implementation researchers often combine the Theoretical Domain Framework (TDF) and Consolidated Framework for Implementation Research (CFIR) in their studies. However there is some debate on the merits of using multiple frameworks—whether they contribute to results or provide superfluous analysis. Our recent research combined the TDF and CFIR to identify determinants to widespread incorporation of patient held medication lists (PHML) in healthcare practice. The aim of this report is to provide guidance on the use of the TDF and CFIR; by assessing the degree of overlap between the two frameworks in their application to interviews about PHML.

**Methods:**

Semi-structured telephone interviews were conducted with healthcare professionals (HCPs) and non HCPs (people taking multiple medicines and caregivers).Interview data were transcribed and analysed using the TDF and CFIR. Within paired domains substantial intersection/overlap across constructs and domains within the two frameworks was classified as > 75% of coding references, consistent intersection/overlap was defined as > 50% and ≤ 75%, average intersection/overlap was defined as ≤ 50% and > 25% and non-substantial intersection/overlap was classified as ≤ 25% of coding references.

**Results:**

Interview data were collected from 39 participants – 21 HCPs and 18 non HCPs.

Mapping of TDF domains to CFIR domains/constructs identified key determinants in six TDF domains: *Environmental context & resources, Beliefs about capabilities, Beliefs about consequences, Social influences, Behavioural regulation* and *Social/professional role & identity*; and five CFIR domains: Intervention Characteristics, Outer Setting, Inner Setting, Characteristics of Individual and Process. A pattern of substantial intersection/overlap in coding emerged with broad TDF domains such as *Environmental context & resources* often linked to well-defined CFIR domains and constructs (e.g. design quality & packaging within Intervention Characteristics). Broad CFIR constructs such as knowledge & beliefs about intervention within Characteristics of Individuals also linked to more descriptive TDF domains like *Beliefs about capabilities*. In addition there was some unexpected non-substantial intersection/overlap in coding with the TDF domain *Social influences* less frequently linked to the CFIR Inner Setting domain and constructs such as networks and communications.

**Conclusions:**

Identifying intersections/overlaps in coding between CFIR and TDF can assist interpretation of findings in implementation research. The strengths of each framework were exploited in a reciprocal process which provided more information to broad/poorly defined domains and enabled identification of implementation determinants and innovation determinants.

**Supplementary Information:**

The online version contains supplementary material available at 10.1186/s43058-023-00466-8.

Contributions to the literature

- It is unclear for many researchers if using multiple frameworks has advantages beyond the application of a single framework.
- Our findings contribute to the goal of data collection within implementation science—to predict outcomes based on setting-level and recipient-level characteristics.
- Combining the TDF and CFIR facilitated identification of implementation and innovation determinants.

## Background

Numerous frameworks have been developed by implementation scientists to guide the implementation of new practices or changing exiting practices in real-world settings. Using theoretical frameworks can generate information on mechanisms of change that can be targeted in interventions [1]. The Theoretical Domains Framework (TDF) and Consolidated Framework for Implementation Research (CFIR) are determinant frameworks which are widely used in implementation science [2, 3]. The TDF is an integrated framework with 33 behavioural theories arranged into 14 construct domains that provides a broad view of cognitive, affective, social, and environmental influences on practices/behaviours [4]. The CFIR is composed of five domains: Intervention characteristics, Outer setting, Inner setting, Characteristics of individuals, and Process. It also provides a list of constructs which can be used to assess key contextual elements—determinants of current practices, potential barriers/facilitators to behaviour change, and evaluation of implementation strategies [3] Both frameworks are widely used in health research but have their limitations [5–7]. Some CFIR constructs are broad—e.g. other personal attributes—and neither the TDF nor CFIR determines the relative importance of its constructs in successful implementation [8, 9].

It has been suggested that combining the TDF and CFIR can assist studies by addressing distinct and multiple conceptual levels – system and individual – and process factors [1, 5]. A review of studies related to healthcare interventions which combined the TDF and CFIR found they could be applied in a variety of study designs—mixed methods, observational and randomized controlled trials [1]. However there are some concerns that using them in combination introduces unnecessary complexity and redundancy to data analysis and interpretation [1].

The Irish Health Service Executive (HSE) National Quality Improvement (NQI) team are devising a national medication safety campaign – the ‘Know Check Ask’ [10]. The purpose of this campaign is to encourage everyone who takes medicines regularly to keep an up to date list of their medicines. To guide the implementation of the campaign, attitudes to and use of patient held medication lists (PHML), among healthcare professionals (HCPs) and non HCPs, were examined in semi-structured phone interviews and key determinants to widespread integration of PHML in healthcare were identified using the TDF and CFIR [11]. The aim of this report is to provide guidance on the use of the TDF and CFIR; by assessing the degree of overlap between the two frameworks in their application to interviews about PHML.

## Methods

Methods are described in detail elsewhere [11]. In brief semi-structured interviews were conducted with HCPs and non HCPs (patients taking multiple medicines and caregivers) with topic guides informed by the TDF and CFIR. Participants were recruited via social media, patient/carer groups and researchers’ contacts. Sampling strata were age, gender and region. The Royal College of Surgeons of Ireland (RCSI) ethics committee provided ethical approval. Interview data were transcribed and analysed using the TDF and CFIR.

### Data analysis

#### Thematic analysis of PHML interview data

Details of the thematic analysis conducted based on the Framework approach, with TDF (12 domain) and CFIR informing the analysis framework have been described previously [11]. In summary an overview of the data set was initially obtained and after familiarisation, investigators (BO’D, CC) independently coded 10% of interviews in the first phase. This was a deductive process with initial coding to the TDF and then the CFIR constructs and domains. Coders applied the frameworks independently and previously coded content was not accessible to them during coding. After comparison and discussion a codebook, guided by recurring themes, was developed. The second phase refined the codebook by continued coding with novel transcripts (a further 8% of interviews). After review the codebook was then deductively applied to the remaining interviews and used to construct a set of thematic charts categorised according to key TDF and CFIR constructs and domains [12]. The software package NVivo 10 was used to analyse the data. The TDF and CFIR analysis is presented in tabular form with sub-themes, themes and illustrative quotes (See Additional file 1).

#### Establishing degree of overlap between TDF and CIFR

The TDF and CFIR constructs and domains were examined to establish common patterns in coding across all the constructs and domains. Interview text that was coded to both a TDF domain and a CFIR construct and domain was identified and classified as an intersection/overlap**.** The frequency and proportion of coding references that intersected/overlapped was established for each individual CFIR construct and domain and each TDF domain using cross-tabulation. Substantial intersection/overlap across constructs and domains within the two frameworks was classified as > 75% of coding references, consistent intersection/overlap was defined as > 50% and ≤ 75%, average intersection/overlap was defined as ≤ 50% and > 25% and non-substantial intersection/overlap was classified as ≤ 25%. The Standards for reporting qualitative research (SRQR) guidelines were adhered to throughout this study [13].

## Results

The interview data about views on PHML included 39 participants – 21 HCPs and 18 non HCPs (patients and carers) – 74% (29/39) were females, median age was 45 years for HCPs (IQR = 37–48), 55 years (IQR = 49–61) for non-HCPs and average number of years of professional practice for HCPs was 18.37 years (SD ± 10.59). (See Table 1). The details of the themes (see Additional file 1) linked to views and attitudes about use of PHML among HCPs, patients and carers of those taking medicines has been previously described [11].

**Table 1:** Characteristics of participants (*n* = 39)

*Demographics*	*Frequency*	*Demographics*	*Frequency*
*Age (years)*		***Gender***	
*Below 40*	*10*	*Female*	*29*
*41–50*	*12*	*Male*	*10*
*51–60*	*10*		
*61–70*	*5*		
*71–80*	*2*		
*Group*		***HCP role***	
*HCP*	*21*	*Doctor*	*8*
*Patient* ^a^	*9*	*Pharmacist*	*9*
*Carer*	*9*	*Nurse*	*4*
*Region*		***Years in practice***	
*East*	*13*	*Doctors*	*5–43 yrs*
*West*	*11*	*Pharmacists*	*9mths-25*
*South*	*15*	*Nurses*	*25–32 yrs*

^a^All patients used PHML; *mths* months, *yrs* years

### Overall mapping of TDF domains and CFIR constructs and domains

**Fig. 1:**
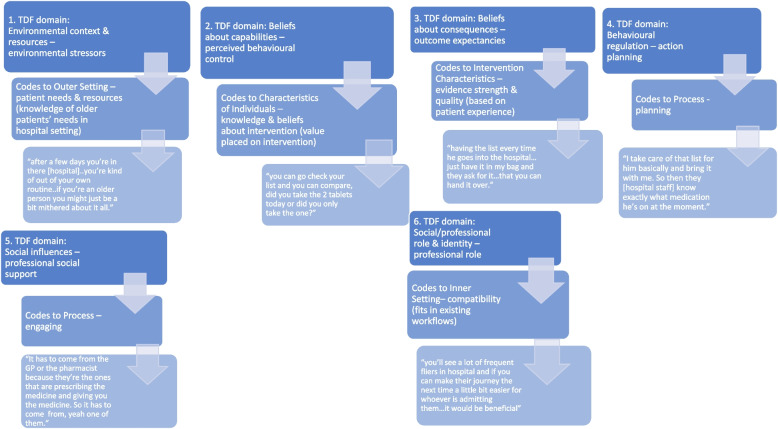
Data excerpts demonstrating intersections in coding between TDF domains and CFIR domains/contructs

**Fig. 2:**
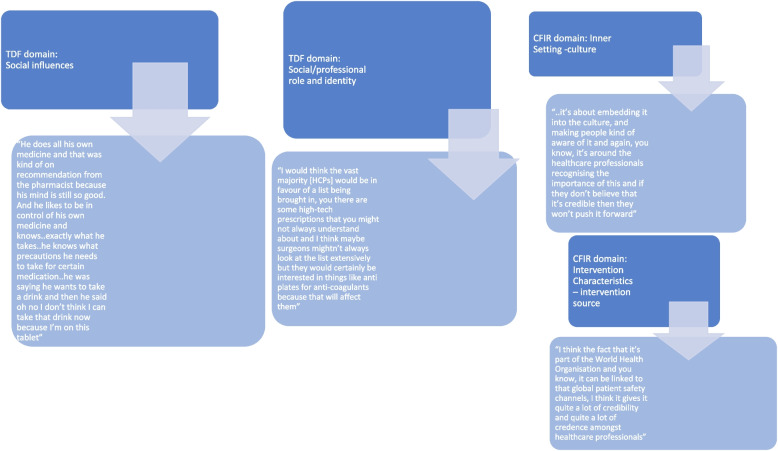
Examples of data excerpts that solely coded to TDF or CFIR domains

Overall key determinants likely to influence use of PHML were previously identified in six key TDF domains: *Environmental context & resources; Beliefs about capabilities; Beliefs about consequences; Social influences; Behavioural regulation* and *Social/professional role & identity;* and five key CFIR domains: Intervention Characteristics (IC); Outer Setting (OS); Inner Setting (IS); Characteristics of Individual (CI); and Process (P). All 39 interviews contained instances of intersection/overlaps; where the same interview text was linked to multiple TDF and CFIR constructs and domains. See Figs. 1 and 2 for data extracts demonstrating coding intersections between the frameworks and extracts that coded solely to TDF or CFIR. A total of 247–600 text blocks were coded across the five key CFIR domains and between 131–578 text blocks were coded across the six key TDF domains. (See Additional file 2 for coding details across TDF and CFIR.)

### Intersections/overlaps in coding between individual CFIR domains and constructs and the TDF domains

In total 382 (81%) coding references within the CFIR domain IC substantially intersected/overlapped with the six key TDF domains. (See Table 2 below.) Seven individual constructs within this domain all displayed substantial intersection/overlapping. For the CFIR domain OS, 184 (74%) coding references in total consistently intersected/overlapped with the six key TDF domains. Two associated constructs—‘External Policies and Incentives’, ‘Patient Needs and Resources’—displayed either substantial or consistent intersection/overlapping. Within the CFIR domain IS 306 (73%) coding references consistently intersected/overlapped with the six key TDF domains and with nine individual constructs. The majority of these constructs (five) consistently intersected/overlapped with four displaying substantial intersection/overlaps in coding. In total 434 (72%) coding references with the CFIR domain CI consistently intersected/overlapped with the six key TDF domains. Three associated constructs – ‘Knowledge and Beliefs’, ‘Individual Identification with the Organisation’, ‘Other Personal Attributes’—displayed substantial intersection/overlapping while one construct ‘Individual Stage of Change’ consistently intersected/overlapped. Within the CFIR domain P 339 (76%) coding references consistently intersected/overlapped with the six key TDF domains. Two individual constructs – ‘Planning’, ‘Engaging’—displayed either substantial or consistent intersection/overlapping. See summary table below with further detail in Additional file 3.

**Table 2:** Summary of intersections/overlaps in coding between individual CFIR and the six TDF domains

Key CFIR domains	*Total coding with key TDF domains*	*Substantial intersection within CFIR constructs and TDF domains*	*Consistent intersection within CFIR constructs and TDF domains*
Intervention Characteristics (IC) (*N* = 474)	382(81%)	Intervention source (5; 100%) (Environmental context & resources (1;20%); Beliefs about capabilities (1;20%); Beliefs about consequences(2,40%); Behavioural regulation (1,20%) Evidence Strength & Quality (84;76%) (Environmental context & resources (11;13%); Beliefs about capabilities (9,11%); Beliefs about consequences (38;45%); Social influences (4,5%); Behavioural regulation (6;7%); Social/professional role & identity (16;19%) Relative Advantage (47;80%) (Environmental context & resources (16;34%); Beliefs about capabilities (7;15%); Beliefs about consequences (15;32%); Social influences (1;2%); Behavioural regulation (7;15%); Social/professional role & identity (1;2%) Adapatibility (102;82%) (Environmental context & resources (37;36%); Beliefs about capabilities (8;8%); Beliefs about consequences (29;28%); Social influences (3;3%); Behavioural regulation (17;17%); Social/professional role & identity (8;8%) Complexity (61;82%) (Environmental context & resources (9;15%); Beliefs about capabilities (7;11%); Beliefs about consequences (25;41%); Behavioural regulation (14;23%); Social/professional role & identity (6;10%) Design Quality & Packaging (78;81%) (Environmental context & resources (37;47%); Beliefs about capabilities (7;9%); Beliefs about consequences (16;21%); Social influences (3;4%); Behavioural regulation (11;14%); Social/professional role & identity (4;5%) Cost (5;100%) (Environmental context & resources (1;20%); Beliefs about consequences (2;40%); Social/professional role & identity (2;40%)	N/A
Outer Setting (OS) (*N* = 247)	184 (74%)	External Policies and Incentives (6;100%) (Environmental context & resources (1;17%); Beliefs about consequences (1;17%); Social influences (1;17%); Behavioural regulation (1;17%); Social/professional role & identity (2;33%)	Patient Needs & Resources (178;74%) (Environmental context & resources (43;24%); Beliefs about capabilities (35;20%); Beliefs about consequences (43;24%); Social influences (8;5%); Behavioural regulation (28;16%); Social/professional role & identity (21;12%)
Inner Setting (IS) (*N* = 419)	306(73%)	Networks & Communication (35;71%) (Environmental context & resources (7;20%); Beliefs about consequences (9;26%); Social influences (5;14%); Behavioural regulation (5;14%); Social/professional role & identity (9;26%) Implementation Climate (66;81%) (Environmental context & resources (16;24%); Beliefs about consequences (15;23%); Social influences (2;3%); Behavioural regulation (15;23%); Social/professional role & identity (18;27%) Organisational Incentives & Rewards (27;82%) (Environmental context & resources (9;33%); Beliefs about consequences (6;22%); Behavioural regulation (2;7%); Social/professional role & identity (10;37%) Available Resources (42;78%) (Environmental context & resources (27;64%); Beliefs about consequences (4;10%); Social influences (2;5%); Behavioural regulation (3;7%); Social/professional role & identity (6;14%)	Culture (27;63%) (Beliefs about consequences (9;33%); Social influences (2;7%); Behavioural regulation (2;7%); Social/professional role & identity (14;52%) Tension for Change (7;54%) (Environmental context & resources (2;29%); Beliefs about consequences (3;43%); Social/professional role & identity (2;29%) Compatibility (42;68%) (Environmental context & resources (1;2%); Beliefs about capabilities (3;7%); Beliefs about consequences (11;26%); Behavioural regulation (13;31%); Social/professional role & identity 14;33%) Relative Priority (20;71%) (Environmental context & resources (4;20%); Beliefs about capabilities (2;10%); Beliefs about consequences (11;55%); Behavioural regulation 3;15%) Access to knowledge/information (40;71%) (Environmental context & resources (20;50%); Beliefs about capabilities (4;10%); Beliefs about consequences (6;15%); Social influences (2;5%); Behavioural regulation (2;5%); Social/professional role & identity (6;15%)
Characteristics of Individuals (CI) (*N* = 600)	434(72%)	Knowledge & Beliefs (278;77%) (Environmental context & resources (29;10%); Beliefs about capabilities (68; 24%); Beliefs about consequences (98;35%); Social influences (20;7%); Behavioural regulation (34;12%); Social/professional role & identity (29;10%) Individual Identification with the Organisation (70;72%) (Environmental context & resources (4;22%); Beliefs about capabilities (1;6%); Beliefs about consequences (4;22%); Social influences (1;6%); Social/professional role & identity (8;44%) Other Personal Attributes (31;91%) (Environmental context & resources (5;16%); Beliefs about capabilities (7;23%); Beliefs about consequences (3;10%); Social influences (2;6%); Behavioural regulation 7;23%); Social/professional role & identity (7;23%)	Individual Stage of Change (70;72%) (Environmental context & resources (2;3%); Beliefs about capabilities (13;19%); Beliefs about consequences (26;37%); Social influences (5;7%); Behavioural regulation (11;16%); Social/professional role & identity (13;19%)
Process (P) (*N* = 448)	339(76%)	Engaging (191;81%) (Environmental context & resources (99;52%); Beliefs about capabilities (5;3%); Beliefs about consequences (18;9%); Social influences (15;18%); Behavioural regulation (16;8%); Social/professional role & identity) (38;20%)	Planning (148;72%) (Environmental context & resources (38;26%); Beliefs about capabilities (17;11%); Beliefs about consequences (19;13%); Social influences (4;3%); Behavioural regulation (45;30%); Social/professional role & identity (25;17%)

### Intersections/overlaps in coding between individual TDF domains and the CFIR constructs and domains

In total 426 (74%) coding references within the TDF domain *Environmental context and resources* consistently intersected/overlapped with the five CFIR domains. (See Table 3 below). There were substantial/intersection/overlap in coding with ‘Patient needs and Resources’ in the CFIR OS domain. This TDF domain consistently intersected/overlapped with two individual constructs – ‘Knowledge and Beliefs’ in the CFIR CI domain and ‘Engaging’ in the CFIR P domain. There were non-substantial intersection/overlap between *Environmental context & resources* and three CFIR domains (IC, IS, CI) and seven associated constructs – ‘Intervention Source’, ‘Cost’, External policy and Incentives’; ‘Compatibility’, ‘Tension for Change’, ‘Relative Priority’ and ‘Individual Stage of Change’. These constructs each contributed ≤ 5% of total coding coverage within the paired TDF and CFIR domains and constructs (See Additional file 3).

**Table 3:** Summary of intersections/overlaps in coding between TDF domains and individual CFIR domains and constructs

Key TDF domains	Total overlap with CIFR domains and constructs	Substantial intersection within CFIR domains and constructs	Consistent intersection within CFIR domains and constructs
Environmental context & resources (*N* = 578)	426(74%)	Patient Needs & Resources (43;98%) (OS)	Knowledge & Beliefs (29;62%) (CI) Engaging (99;72%) (P)
Beliefs about capabilities (*N* = 332)	201(61%)	Patient Needs & Resources (35;100%) (OS) Planning(17;77%) (P)	Knowledge & Beliefs (29;62%) (CI)
Beliefs about consequences (*N* = 528)	422(80%)	Patient Needs & Resources (43;98%) (OS)	Knowledge & Beliefs (98;70%) (CI) Planning (19;51%) (P)
Social influences (*N* = 131)	80(61%)	Patient Needs & Resources (8;89%) (OS) Engaging (15;79%) (P)	Knowledge & Beliefs (20;71%) (CI)
Behavioural regulation (*N* = 408)	250(61%)	Patient Needs & Resources (28;97%) (OS)	Knowledge & Beliefs (34;58%) (CI) Planning (45;74%) (P)
Social/professional role & identity (*N* = 363)	266(73%)	Patient Needs & Resources (21;91%) (OS)	Engaging (38;60%) (P)

For the TDF domain *Beliefs about capabilities*, 201 (61%) of total coding references consistently intersected/overlapped with the five CFIR domains. This TDF domain substantially intersected/overlapped with ‘Patient Needs and Resources’ within the CFIR OS domain and ‘Planning’ within the P domain. It also consistently intersected/overlapped with ‘Knowledge and Beliefs’ in the CFIR CI domain. *Beliefs about capabilities* did not code with six individual constructs within the IS domain and there was also non-substantial intersection/overlap between this TDF domain and twelve associated constructs. Three CFIR constructs—‘Identification with Organisation’, ‘Self-efficacy’ and ‘Other Personal Attributes’—in the CI domain and ‘Intervention Source’ within the IC domain each contributed ≤ 7% of total coding coverage.

For the TDF domain *Beliefs about consequences* 422 (80%) of total coding references substantially intersected/overlapped with the five CFIR domains. There was substantial intersections/overlaps between this TDF domain and ‘Patient Needs and Resources’ in the CFIR OS domain. It also consistently intersected/overlapped with two individual CFIR constructs—‘Knowledge and Beliefs’ and ‘Planning’ in the CI and P domains respectively. There were non-substantial intersections/overlaps in coding with twenty associated constructs with six of these constructs each contributing ≤ 3% of total coding coverage.

In total 80 (61%) coding references within the TDF domain *Social influences* consistently intersected/overlapped with the five CFIR domains. This TDF domain substantially intersected/overlapped with ‘Patient Needs and Resources’ within the OS domain and ‘Engaging’ within the P domain. It also consistently intersected/overlapped with ‘Knowledge and Beliefs’ in the CI domain. *Social influences* did not code with seven individual constructs within the CFIR domains and there was also non-substantial intersection/overlap between this TDF domain and ten associated constructs.

Within the TDF domain *Behavioural regulation* 250 (61%) coding references consistently intersected/overlapped with the five CFIR domains. There was substantial intersections/overlaps with ‘Patient Needs and Resources’ in the OS domain and consistent intersections/overlaps with ‘Knowledge and Beliefs’ and ‘Planning’ in the CI and P domains respectively. *Behavioural regulation* did not code with three constructs—‘Cost’ (IC domain), ‘Tension for Change’ (IS domain) or ‘Identification with Organisation’ (CI domain).

The TDF domain *Social/professional role and identity* 266 (73%), consistently intersected/overlapped with the five CFIR domains. There was substantial intersections/overlaps in coding with ‘Patient Needs and Resources’ in the OS domain and consistent intersections/overlaps with ‘Engaging’ in the P domain. *Social/professional role and identity* did not code to ‘Intervention Source’ or ‘Relative Priority’ in the IC and IS domains respectively. There was non-substantial intersection/overlapping with eighteen CFIR constructs with three of these constructs each contributing ≤ 9% of total coding coverage within the paired TDF and CFIR domains and constructs (‘Cost’, ‘Tension for Change’ and ‘External Policy and Incentives’). (See summary table, with further detail in Additional file 3).

## Discussion

This study found it beneficial to combine the TDF and CFIR to address the limitations of the two frameworks identified by previous research such as broad or poorly defined domains, variation in which domains/constructs are used, gaps between determinants and outcomes [3, 5, 14]. In general all of the coding related to a similar theme/idea and mapping of TDF domains to CFIR constructs resulted in some intersecting/overlapping between TDF domains and CFIR domains and constructs. Some of these patterns may be anticipated i.e. overlaps between *Beliefs about consequences* and ‘Knowledge and Beliefs about intervention’; divergence between *Behavioural regulation* and ‘Cost’. In particular, this study identified intersections/overlaps in coding with some specific reciprocal benefits. Broad TDF domains such as *Environmental context & resources* displayed substantial overlaps with well defined CFIR constructs; while broad CFIR constructs such as ‘Knowledge and Beliefs about intervention’ (within CI) were linked to more elaborate/descriptive TDF domains such as *Beliefs about capabilities*. Our findings suggest that the frameworks were often complimentary; combining the TDF and CFIR can lead to additional information on key determinants such as individual characteristics, context, resources and result in increased targets for implementation efforts [8, 15].

Implementation research has suggested that overarching frameworks like the TDF can be augmented with broader frameworks such as the CFIR which can address contextual issues/barriers e.g. practical access to resources [15]. In this study, as might be expected, the CFIR diverged from the TDF in identifying organisational determinants such as ‘Tension for Change’ and practical issues such as intervention design/incentives. The overall pattern of substantial/non-substantial coding of CFIR domains and constructs could indicate context-specific determinants where constructs such as ‘Tension for Change’ or ‘External Policy and Incentives’ may have a more direct impact than intervention/implementation costs. There was also divergence between the TDF domain *Social*/*professional role & identity* and some CFIR constructs associated with organisational receptivity to intervention. This pattern of non-substantial intersection/overlapping in coding may indicate that organisational factors could exist in a hierarchy which mediates perceptions and influences HCPs to varying degrees. Prioritising constructs with substantial intersections/overlaps in coding, such as ‘Implementation climate’ when selecting targets for interventions could prove to be beneficial [16].

In general the frameworks were complementary however some non-substantial intersections/overlaps between TDF and CFIR emerged in the area of social support. It could be anticipated that TDF domains related to social support/norms would frequently map to equivalent CFIR domains and constructs associated with structured support at an organisational level such as ‘Networks and Communications’, ‘Culture’, ‘Peer Pressure’ and ‘Champions’. However non-substantial coding between the frameworks in this area could indicate that TDF provides more precise coding of social support than CFIR and that CFIR may identify structured social influences but possibly capture less information about social networks. Using the TDF ensures that the impact of social relationships at the individual level; which may vary across cultures and/or ethnicities; are thoroughly investigated [17, 18].

Combining the TDF and CFIR had numerous benefits; increasing the depth and breadth of relevant information through complementary processes that provide useful information to broad/poorly defined domains. However, it should be noted that applying both frameworks to interview data had implications for project management and timelines. The iterative process of double-coding required considerable expenditure of research time and resources.

A limitation of this study was the possibility of bias – selection and/or researcher. However attempts were made to address these issues with purposive sampling of participants; to achieve a range of clinical experience and health conditions. Methodological strategies were also employed to reduce researcher bias; mapping decisions were independently made and reviewed to ensure robust alignment of mappings [19]. Sequential coding – first TDF and then CFIR—was conducted for time management purposes as coders had extensive experience with the TDF. It should be noted that results may have differed if a different sequence was employed or both frameworks were applied simultaneously.

Recent CFIR research has added outcomes to the CFIR framework, assessing implementation (setting-level) and innovation (recipient-level) determinants to explain/predict outcomes [20]. Implementation determinants are within the scope of the CFIR however innovation determinants are theory driven and the CFIR was not designed to capture these [20]. A theoretical framework such as the TDF, which is based on multiple behavioural change theories, would be useful in capturing recipient-level characteristics and experiences. Combining the TDF and CFIR frameworks could benefit studies, expanding analysis beyond that of a single framework.

## Conclusion

Our findings should be viewed as an initial attempt to systematically record intersections/overlaps in coding with the CFIR and TDF and clarify the value of their combined use in identifying key determinants. Identifying patterns can increase interpretation of findings and create a more robust picture of the implementation environment. This mapping will need continued refinement and validation to establish the benefits of combining these frameworks in implementation research. Future research could examine a greater number of cases to verify coding patterns across the TDF and CFIR.

### Supplementary Information

**Additional file 1.** CFIR & TDF analysis: subthemes, themes & illustrative quotes.

**Additional file 2.** Total coding references & classification within key TDF domains & CFIR domains.

**Additional file 3.** Frequency mapping of key TDF domains to key CFIR domains & constructs.

## Data Availability

Data not publicly available due to privacy or ethical restrictions.

## Abbreviations

EBI: Evidence based interventions
TDF: Theoretical Domain Framework
CFIR: Consolidated Framework for Implementation Research
PHML: Patient-held Medication List
HCPs: Healthcare professionals
NQI: National Quality Improvement team
