# Supplementary material for: Mapping the Theoretical Domain Framework to the Consolidated Framework for Implementation Research: do multiple frameworks add value?
Source: Implement Sci Commun. 2023 Aug 24;4:100. doi: 10.1186/s43058-023-00466-8 (PMC10464139; doi:10.1186/s43058-023-00466-8)
Supplement: Supplementary file 1 — Additional file 1. CFIR & TDF analysis: subthemes, themes & illustrative quotes. [file 43058_2023_466_MOESM1_ESM.pdf]

Additional file 1: CFIR & TDF analysis: subthemes, themes & illustrative quotes

Themes & related subthemes	CFIR domains & constructs	TDF domains	Illustrative quotes
**<u>Attitudes to patient held lists:</u>**<br>Positive attitudes<br>- empowering<br>- increase adherence/awareness<br>- reduce errors/unused meds<br>- aid to patient/HCP communication across healthcare setting, transitions of care, while travelling<br><br>Negative attitudes<br>- concerns about accuracy<br>- concerns about stigma<br>- visual cues used by HCPs to assess lists	Intervention characteristics - *design quality & packaging*, *adaptability*<br><br>Characteristics of individuals - *knowledge & beliefs about intervention;*<br><br>Outer setting – *patient needs & resources*	Environmental context & resources;<br><br>Beliefs about consequences;<br><br>Beliefs about capabilities;<br><br>Professional role & identity.	<u>Positive attitudes – psychological benefits</u><br>*"it's kind of empowering the person to actually have some self-determination on their, you know, on the drugs they're on and to understand why they're on the drugs, and to understand like, you know, as well that it's important for them to have a record of it on their person" (DS30076, hospital nurse)*<br><br><u>Positive attitudes – practical benefits</u><br>*"certainly for out of hours..it makes a big difference..very often sometimes like that in out of hours setting, they're out of their comfort zone..So I think definitely for out of hours and for if somebody had to go to hospital" (DS300065, GP)*<br><br>*"I was traveling extensively, I mean on my holidays..So I always kept, I was always aware that I needed to have something that I could produce to somebody." (DS300054, patient)*<br><br><u>Negative attitudes</u><br>*"sometimes the ones they pull out of their wallet is 2 years old. And in the meantime we have changed things..they can be helpful but they can be very inaccurate." (DS300059, GP)*<br><br>*"certainly if there's like misspellings and that you'd have to have some doubts as to whether, you know, there are other errors…Like we're all influenced by presentation so if something is presented nicely and well done and thought and effort has gone into it and it's been kept well then, you know, that creates a better impression" (DS300044, pharmacist)*
**<u>Function and preferred features of patient held lists:</u>**<br>- variation in use	Intervention characteristics - *design quality & packaging*	Knowledge;<br><br>Beliefs about consequence;	<u>Variation in use</u><br>*"I know that each time my colleagues say what are you on, and they don't have the list." (DS300071, GP)*

- evolving lists - customisation, multiple versions,<br>- varied information needs related to content and detail<br>- HCP support (routine practice)	Characteristics of Individuals - *knowledge & beliefs about intervention*<br><br>Inner setting – *implementation climate (compatibility)*<br><br>Outer setting – *patient needs & resources*	Beliefs about capabilities;<br><br>Memory attention & decision processes;<br><br>Skills;<br><br>Nature of the behaviours	*"Well I was with a consultant there about a week ago and I gave him the list and he was delighted"*<br>*(DS300054, patient)*<br><br><u>Customised lists</u><br>*"I have it in the phone, I also have, you know the little stick on labels that the pharmacist puts on the pack, I have all those on a piece of paper, folded up and its in my wallet as well. And I also keep the copy of the prescription in my folder here at home" (DS300061, patient)*<br><br>*"normally 99% of the times it's a print out from my GP with a list of my meds, all of my meds on it and I photograph it, so its in my photographs. I've also printed off the photograph and cut it down to size and its actually stuck on to my phone as well." (DS300062, patient)*<br><br><u>Multiple lists</u><br>*"So I have to carry a list everywhere, I have one in my bag and one in the car. If I was ever stuck they're there" (DS300064, patient)*<br><br><u>HCP support</u><br>*"every time patients come in, especially elderly, kind of complicated patients we generally would go through their medications with them all the time anyway" (DS300065, GP)*<br><br>*"…an automatic thing if you're doing a prescription for a patient..particularly a repeat prescription…I take that as the cue" (DS300056, GP)*

**Barriers & facilitators to future use of lists:**<br>Barriers<br>- lack of time<br>- difficult for older patients, those with cognitive impairments, literacy issues, multiple/changing medicines<br>- perceived reticence among older patients<br>- generic medicines cause of confusion<br>- concerns about privacy*<br>- lack of engagement about PHML from HCPs*<br>Facilitators<br>- role for trusted HCPs (pharmacists, GPs, public health nurses)<br>- practical tools e.g. compact (wallet-sized version) or digital options (phone app)<br>- self efficacy[#]<br>- internal & external strategies** e.g. routine behaviour, medicine lists, blister packs*, memory aids (phone apps)<br>- family support**<br>- use of simple language by HCPs[#]<br>- regular medication reviews for specific	Intervention characteristics - *design quality & packaging*<br><br>Characteristics of Individuals - *knowledge & beliefs about intervention*, *self efficacy*<br><br>Inner setting – *implementation climate (compatibility, relative priority)*<br><br>Outer setting – *patient needs & resources*<br><br>Process - *engaging*	Environmental context & resources;<br><br>Beliefs about capabilities;<br><br>Social influences;<br><br>Behavioural regulation;<br><br>Professional role & identity	**Barriers**<br>HCPs' lack of time<br>*"you could ask to talk to the pharmacist and they're so busy that they can't talk to you" (DS300064, patient)*<br><br>Difficulties for older patients<br>*"some older patients..a lot of them would be on polypharmacy, not all of them but most of them, some of them aren't tech savvy" (DS300065, GP)*<br><br>Reticence in questioning HCPs<br>*older people just think well the doctor knows..they just see the doctor as god" (DS300079, carer)*<br><br>Confusion associated with generics<br>*"sometimes, they'll [his tablets] change in name and that can be very confusing actually, these generic medications where the names are changing, that's a big thing now I just find that's really difficult" (DS300051, carer)*<br><br>Lack of engagement with carers from HCPs<br>*"I think that there's no information about this [lists] coming from the GPs or the public health nurses or even from the hospitals…none of this information is passed on from any of those people." (DS300084, carer)*<br><br>**Facilitators**<br>Role for key HCPs<br>*"It has to come from the GP or the pharmacist because they're the ones that are prescribing the medicine and giving you the medicine." (DS300051, carer)*<br><br>*"I think pharmacists have a really big part to play in it" (DS300046, patient)*<br><br>*"That's our role. We are supposed to counsel and we are supposed to manage medication" (DS300044, pharmacist)*<br><br>Digital tools

patients (older/multiple medicines)<br>- medication counselling[##]			*"I'd love something like that [phone app] because, well most people now live by their phone don't they really, it's all apps, everything is on an app. I'd love it." (DS300051, carer)*<br><br>Routine behaviour<br>*"So you know when we are sitting down having a cup of tea or something at the table that's when, like I never forget taking my tablets. Just got used to it now simple as that" (DS300063, patient)*<br><br>Memory aids<br>*"I've an app on the phone that reminds me to take it" (DS300078, patient)*<br><br>Blister packs*<br>*"I think they're fantastic because there can be no mistakes made with medication when its blister packed…You couldn't make a mistake if you tried" (DS300085, carer)*<br><br>Family support**<br>*"I've always had a list in the house for him…we're always coming in and out so..if they ever need it I'd have a list there in the house stuck up on the wall" (DS300051, carer)*<br><br>Regular medication reviews[##]<br>*"one time they had a pain in their big toe and they were prescribed a painkiller and that suddenly stayed in their regular prescription. So they have bottles and bottles and bottles of paracetamol which they probably take once every six months…so they're definitely is room there for review for medications."*<br>*(DS300073, GP Nurse)*

*reported by carers; **reported by patients & carers; [#]reported by patients; [##]reported by HCPs
